# Tannic Acid-Loaded Antibacterial Hydroxyapatite-Zirconia Composite for Dental Applications

**DOI:** 10.3390/cryst15050396

**Published:** 2025-04-24

**Authors:** Nusrat Yeasmin, Joel Pilli, Julian McWilliams, Sarah Norris, Arjak Bhattacharjee

**Affiliations:** 1 Sustainable Manufacturing and Tissue Engineering Laboratory, Department of Materials and Metallurgical Engineering, New Mexico Institute of Mining and Technology, Socorro, NM 87801, USA; 2 Department of Biology, New Mexico Institute of Mining and Technology, Socorro, NM 87801, USA; 3 Department of Biochemistry, Tufts University, Medford, MA 02155, USA; 4 Department of Biology, Tufts University, Medford, MA 02155, USA

**Keywords:** antibacterial, tannic acid, dental materials, zirconia, hydroxyapatite

## Abstract

The development of advanced biomaterials for dental applications has gained significant attention due to the need for enhanced mechanical properties, biocompatibility, and antibacterial activity. Hydroxyapatite (HA) is widely used in bone tissue engineering owing to its chemical similarities to bone. However, biofilm formation and bacterial infection on HA may lead to implant failure and revision surgery. Tannic acid, a polyphenolic compound with strong antibacterial and antioxidant properties, was incorporated into the composite to provide antimicrobial effects, that may address the challenge of biofilm formation on dental surfaces. In this study, the biomedical potential of tannic acid (TA)-loaded hydroxyapatite-zirconia composites were analyzed. The crystallization characteristics, functional groups, and morphology were analyzed using X-ray diffraction (XRD), Fourier transform infrared spectroscopy (FTIR), and field emission scanning electron microscopy (FESEM) analysis. The biocompatibility of composite samples was analyzed through *in vitro* cell culture studies. The combined effect of TA and zirconia showed antibacterial efficacy against *Staphylococcus aureus* (*S. aureus*) after 24 h of sample–bacterial interactions. The results demonstrate that this tannic acid-loaded hydroxyapatite-zirconia composite holds significant promise for improving the performance of dental materials and preventing infections in oral healthcare applications.

## 1. Introduction

Bone disorders are increasingly common, leading to a rise in the demand for bone reconstruction [1,2]. Due to the modern lifestyle of younger adults, the demand for bone reconstruction in younger patients is rising globally. Traditional treatments for bone disorders typically involve high doses of chemotherapeutic drugs and surgical intervention [3,4]. Moreover, bacterial growth on the implant surface and biofilm formation is a frequent complication that involves the cultivation of bacteria at the surface of the bone graft material within the oral cavity, often leading to implant failure and requiring revision surgery [5–7]. These bacterial adhesions may result in inflammation, infection, and eventually, the necessary removal of implants. Once this biofilm has formed, the site may be resistant to these antibiotics [8]. Biofilms are particularly challenging to treat because of their complex DNA and protein structure, which prevents antibiotics from effectively penetrating the biofilm, leaving patients susceptible to infections that compromise the success of the dental implant. The treatment of bacterial colonization on the surface of the implant is complex to specifically target and treat considering this resistance to penetration of the biofilm. Current bone grafting techniques highlight the need for intrinsic antibacterial properties of the materials to prevent negative consequences of infection. Also, it is required to develop artificial bone implants with increased longevity.

In bone tissue engineering, implants are widely used for repairing injured bones or teeth. From the 2022 Global Oral Health Report of the World Health Organization (WHO), it is found that globally, around 3.5 billion people suffer from oral diseases [9]. Dental implants are widely used in dentistry to replace missing teeth because these are made of lightweight but strong material that withstands the forces of chewing and fuses firmly to the jawbone, providing a solid foundation [10,11]. The success of implant treatment and its life-time depends primarily on the materials in the implant components [10]. An ideal material to be used for dental implant and restoration applications should have high compressive strength, hardness, wear resistance, chemical resistance, and biocompatibility [10,12]. The implant material should also be aesthetically consistent with natural teeth [10]. Thus, in bone grafting, bioceramic materials are used because of their compatibility with biological tissues, chemical inertness, non-toxicity, and mechanical properties [10,13].

Hydroxyapatite [HA, Ca_10_(PO_4_)_6_(OH)_2_] is a biomaterial that carries similar properties in terms of biocompatibility, bioactivity, and chemical similarities to bone [14–16]. HA is naturally present in the human body and serves as the primary structural element of tooth enamel and bone minerals. In tissue engineering, HA is widely used in dental bone grafting and augmentation in the mouth as well as maxillofacial surgery because HA facilitates better integration, allowing bone cells to attach, grow, and proliferate on their surface and heal [15]. However, HA is brittle and thus cannot provide the same mechanical properties and benefits as natural bone. From this, HA has lower mechanical properties and an increased rate of degradation, which limits its application for practical uses [5,14]. It lacks inherent antibacterial properties on its own. Thus, the need for incorporation of the drug into the bone graft is imperative. [5]. Scientists can either dope the HA with various ions or make HA-based composites to modify the mechanical and biological properties to increase the usability of HA [5,17]. Composite can be made with metal ions like silver, zinc, and copper ions to attain the desired antibacterial effects to the HA framework [5]. Increasing the mechanical properties allows HA to be used for greater load bearing, increasing the uses of HA as a bioactive ceramic because it can fuse with bone tissue and promote healing. HA-based composite making with a similar goal is also another option. The primary scientific question addressed in this study is: “Is it possible to use HA-zirconia composite combined with plant-derived tannic acid (TA) to create an alternative antibacterial and cytocompatible scaffold for bone tissue engineering and dental applications?”.

Zirconia (ZrO_2_), a transition metal oxide, is widely used in dentistry because of its outstanding mechanical, bio-inert properties, low reactivity, non-toxicity, and biocompatibility [5,15,18]. The addition of zirconia to hydroxyapatite increases the material’s durability and strength, which is crucial for the material’s performance in dental applications that involve high-stress environments, such as implants, crowns, and fillings [5,14,19]. The addition of zirconia enhances the structural integrity of hydroxyapatite, ensuring longevity and durability [5]. ZrO_2_ may lower bacterial adhesion. The impact of ZrO_2_ on improving the mechanical and biological properties of HA has been investigated by many researchers in dental and orthopedic fields [20–22]. One previous work found that cerium oxide and zirconium oxide can individually show antibacterial properties against the *S. aureus* and *E. coli* bacteria [5]. Another work reported that 40% Zr^4+^ and 60% Zr^4+^ in HA are widely used in the biomedical field [5]. In a previous work, the researchers created ZrO_2_–calcium phosphate composites that exhibited increased strength and toughness, as well as strong biocompatibility [20]. The use of zirconia in these HA models has been proven effective in hardening the material as well as providing antibacterial properties.

Tannic acid (TA) is a compound made up of polyphenols known for its diverse therapeutic properties, including strong antioxidant, antimutagenic, and antibacterial effects. TA has been shown to penetrate bacterial cell walls and disrupt internal cellular functions, leading to bacterial death [23]. It also inhibits the uptake of essential nutrients like sugars and amino acids in bacteria, which further impedes bacterial growth [23]. This study aims to develop a novel TA-loaded HA-zirconia composite that will be inherently antibacterial. Our strategy is to use an alternative antibacterial compound, TA, to address this problem. TA is antibacterial, antioxidant, and anti-inflammatory. In this research, we will use TA as a natural medicinal compound as an alternate strategy to prevent infection on the implant surface.

We hypothesize that the combined effects of TA and zirconia will improve antibacterial performance while maintaining cytocompatibility. We examined the impact of TA-loaded different percentages of HA-zirconia composite on the mechanical and antibacterial properties of the composites. The resulting composites were examined using X-ray diffraction (XRD), field emission scanning electron microscopy (FESEM), Fourier transform infrared spectroscopy (FTIR), cell culture, and antibacterial study. The integration of tannic acid (TA) and zirconia into hydroxyapatite (HA) is being explored as a promising strategy to enhance the antibacterial properties without compromising the mechanical strength of materials used in dental applications. By reducing bacterial viability and adhesion on dental surfaces, TA can help reduce the risk of bacterial infections. TA enhances the overall antibacterial effect when combined with HA-zirconia composite. The dual mechanism of action from both the tannic acid and zirconia enhances the antibacterial protection over conventional HA materials. Therefore, we expect that TA-loaded HA-zirconia composite will exhibit enhanced biological properties. As a novel strategy, we loaded TA with HA-zirconia composite. Therefore, the uniqueness of this study lies in enhancing the antibacterial properties of the scaffolds with acceptable mechanical properties without introducing any toxicity.

## 2. Materials and Methods

### 2.1. Sample Preparation

The HA-zirconia composite was made by mixing commercial hydroxyapatite (HA) powder (NEI, Herrin, IL, USA), and 5% and 20% of zirconia (Alfa Aesar, Haverhill, MA, USA), in ethanol media followed by 2 h of ball milling [2]. As Ca^2+^ ion will be replaced by Zr^4+^ ion, the expected chemical formula might be [Ca_9.6_Zr_0.4_(PO_4_)_6_(OH)_2_] and (Ca_8.4_Zr_1.6_(PO_4_)_6_(OH)_2_) for 5 wt.% and 20wt.% HA-zirconia composite, respectively. We selected the zirconia amount to make composite and biological results according to previous work [2,24–26]. Following ball milling, the samples were dried and subsequently pressed uniaxially for 3–4 min with a hydraulic press. An electric furnace was used to sinter the pressed powder at 1250 °C with 2 h holding. For clarity, the pure HA will be referred to as HA, the 5% HA-zirconia composite will be denoted as HA+5Z, and the 20% HA-zirconia composite will be referred to as HA+20Z.

### 2.2. Assessment of Densification and Dimensional Shrinkage

Following sintering, densification was evaluated by measuring both the samples’ theoretical density and bulk density. Volume shrinkage was determined by measuring the dimensional changes in volume before and after sintering [2]. Radial and longitudinal shrinkage were assessed by measuring the changes in diameter and height before and after sintering. These measurements were performed on three samples to calculate an average and enhance the accuracy of the results [27].

### 2.3. Analysis of Phase and Microstructure

X-ray diffraction (XRD) analysis of both HA-zirconia composite and pure HA samples was conducted using a PANalytical Empyrean instrument over a range of 20° ≤ 2θ≤ 70° to investigate the phase characteristics of both control and HA-zirconia composites [2]. The measurements were performed using a step size of 0.015° (400 s/step), utilizing Cu-Kα radiation with a wavelength of 1.54 Å, at a voltage of 45 kV and a current of 40 mA within the 2θ range of 20° to 70°. This study utilized a Fourier transform infrared spectroscopy (FTIR) using a Nicolet Is50 FTIR instrument (Thermo Scientific, Waltham, MA, USA). The analysis was conducted in the 400–1200 cm^−1^ range for HA, 5% HA-zirconia composite, and 20% HA-zirconia composite, and in the 500–4000 cm^−1^ range for characterizing the functional groups of tannic acid (TA) [2]. The microstructures of the HA-zirconia composites were analyzed utilizing field-emission scanning electron microscopy (FESEM) at a voltage of 15 kV and a magnification of ×5000 [2]. To enhance the surface conductivity, the samples were sputter-coated with platinum. Morphological changes in the HA-zirconia composites were assessed by comparing the FESEM images of HA and HA-zirconia composites.

### 2.4. Assessment of Mechanical Properties

A compressive strength measurement was performed to assess the structural characteristics of the control HA sample and HA-zirconia composite. The compressive strength test was conducted using a hydraulic press. This test is a critical step in evaluating the mechanical properties of scaffolds. The breaking point of the samples at the highest load was used to determine the stress as load per unit area and compared with the control and HA-zirconia composite. After getting compressive strength in N/m^2^, we have converted it to Pa and then MPa.

(1) $$\text{Compressive}\;\text{Strength}\left({\text{N/m}}^{\text{2}}\right)=\text{Maximum}\;\text{Load}\left(\text{N}\right)/\text{Cross-sectional}\;\text{area}\left({\text{m}}^{2}\right)$$

### 2.5. Tannic Acid Loading and Characterization

An ethanolic solution of TA at a concentration of 7 mg/mL was made and loaded at specific quantity on the upper layer of each scaffold to assess its cytocompatibility and antibacterial properties. The functional groups of TA were analyzed using FTIR spectra within the 500–4000 cm^−1^ range.

### 2.6. Antibacterial Efficacy Assessment

#### 2.6.1. Modified ISO 22196:2011 Standard

The antibacterial activity of both TA-loaded HA-zirconia composite and pure HA samples was evaluated for activity against gram-positive bacteria *Staphylococcus aureus* (*S. aureus*) following a modified version of the ISO 22196:2011 Standards, as described in prior research [28]. The samples were sterilized using an autoclave, after which TA was loaded onto the surface of each sterilized sample. The freeze-dried bacterial stock was then activated following the supplier’s recommended procedure. The optical densities of bacterial suspensions at various concentrations were assessed using a UV–VIS spectroscopy microplate reader and compared with the McFarland standard [28–30]. The sterilized samples were transferred to 24-well plates, and 10^5^ CFU of bacteria was pipetted on top of each sample. Afterward, 1 mL of broth media was introduced, followed by plates being incubated at 37 °C for 24 h. Following incubation, the samples were transferred to a glass vial. After that, 1 mL of phosphate buffer solution (PBS) was mixed with the samples, and the samples were vortexed for 15 s. Serial dilutions of the vortexed solution were then performed, and 10 μL of the vortexed and diluted solution was applied to agar plates using the streaking method. The plates were incubated at 37 °C for another 24 h, after which bacterial colonies were counted using photographic analysis. The antibacterial effectiveness was determined as 100—bacterial cell viability (%), where bacterial cell viability (%) was determined using the following equation:

(2) $$\text{Bacterial}\;\text{cell}\;\text{viability}\left(\%\right){=\text{X}}_{\text{treatemnt}}{/\text{X}}_{\text{control}}\times 100\%$$

#### 2.6.2. Morphological Characterization

The samples were fixed with 2% paraformaldehyde and 2% glutaraldehyde, refrigerated overnight at 4 °C, and rinsed with 0.1 M PBS. After that, we carried out ethanolic dehydration three times in different concentrations of 30%, 50%, 70%, 95%, and 100% [28,29]. Following dehydration, Hexamethyldisilazane (HMDS) was applied to each sample, which was then placed in a fume hood overnight for drying. We coated the surface with platinum using a sputter coater. Finally, bacterial morphology was analyzed using field-emission scanning electron microscopy (FESEM) to observe the cellular morphology of the samples.

### 2.7. Assessment of Cytocompatibility

#### 2.7.1. Cell Seeding on the Sample Surfaces

Cell–material interaction study was conducted using the NIH3T3 fibroblast cell line (ATCC, Manassas, VA, USA) to evaluate the cytocompatibility effects of zirconia and TA loading. For the cell culture experiments, Dulbecco’s Modified Eagle’s medium (DMEM) supplemented with 10% fetal bovine serum and 1% penicillin/streptomycin was used. After autoclaving and drug loading, the sterilized samples were placed in 24-well plates. Approximately 30,000–35,000 cells were plated onto each sample, followed by 1 mL of culture media. The plates were then incubated in a 5% CO_2_ atmosphere at 37 °C for 24 h [31].

#### 2.7.2. Cell Viability Quantification by MTT Assay

Cell viability was examined using the 3-(4,5-dimethylthiazol-2-yl)-2,5-diphenyl tetra-zolium bromide (MTT) assay after cell-material interactions. The samples were moved to new well plates, and 100 μL of MTT solution, along with 900 μL of media, were incorporated, followed by incubation at 37 °C for 2 h. After incubation, the media was replaced with 600 μL of MTT solubilizer, and 100 μL of the resulting solution was pipetted to a 96-well plate. The optical density was measured using a UV–VIS microplate reader to determine cell viability, with biological experiments conducted in triplicate [32,33].

## 3. Results

**Figure 1.**
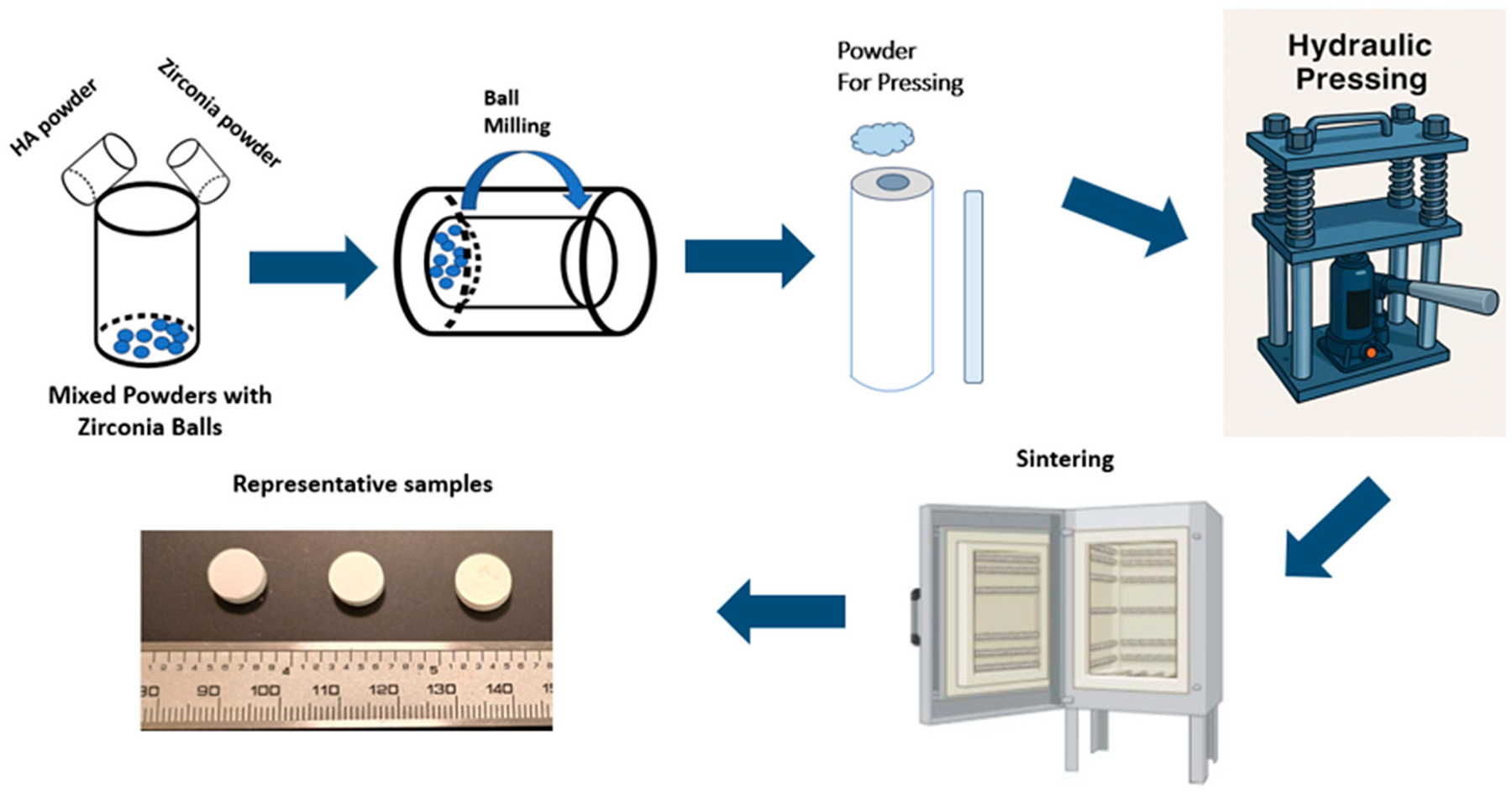
Diagram illustrating the sample preparation process, beginning with the mixing of hydroxyapatite (HA) with zirconia, followed by ball milling, hydraulic pressing, and sintering. Images representing the resulting samples are also shown.

In Figure 1, a schematic representation of HA-zirconia composite via ball milling followed by hydraulic pressing and sintering is shown. The selected sintering temperature employed was 1250 °C. After sintering the bulk density (g/cm^3^), the volume, radial, and longitudinal shrinkage (%) are displayed in Table 1. The bulk densities were 2.66 g/cm^3^ for HA, 2.72 g/cm^3^ for 5% HA-zirconia composite, and 2.03 g/cm^3^ for 20% HA-zirconia composite. HA had a volume, radial, and longitudinal shrinkage of 55.67%, 23.75%, and 21.78%, respectively; for 5% HA-zirconia composite they were: 57.77%, 24.97%, and 24.48%, respectively; for 20% HA-zirconia composite they were: 42.74%, 16.96%, and 16.39%, respectively.

**Figure 2.**
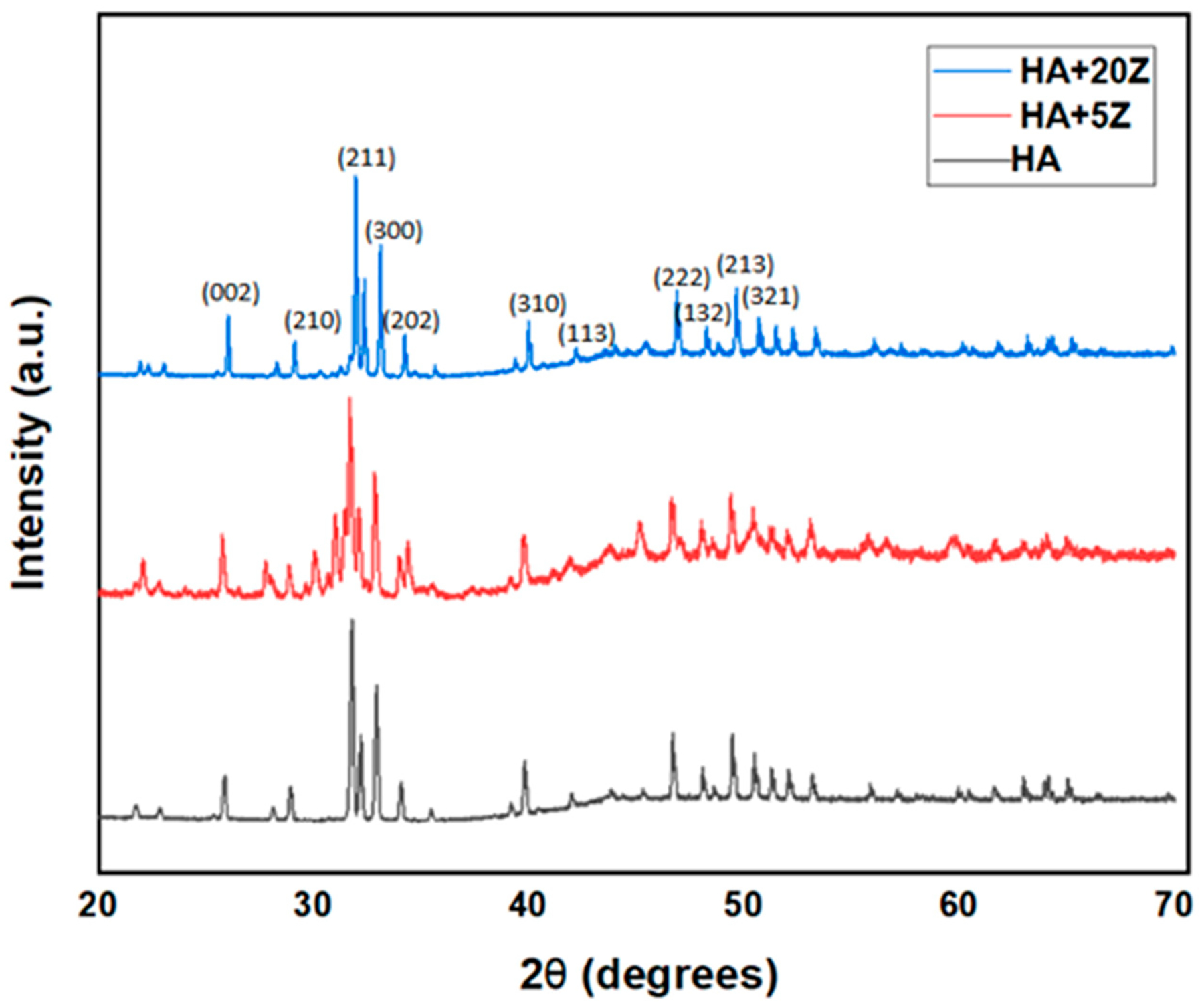
XRD patterns of HA-zirconia composite with 5% and 20% zirconia, as well as pure HA, are presented in the 20–70° range. Some characteristic peaks are identified and labeled in the figure.

Figure 2 depicts the XRD results of all sintered samples in the range of 20°–70°. The standard peaks are observed and marked in Figure 2 [34]. From JCPDS #09–0432, the HA phases are confirmed [30]. These confirms the purity of HA [2,20,35–39]. Retention of HA peaks are noticed in the HA-zirconia composites as well.

**Figure 3.**
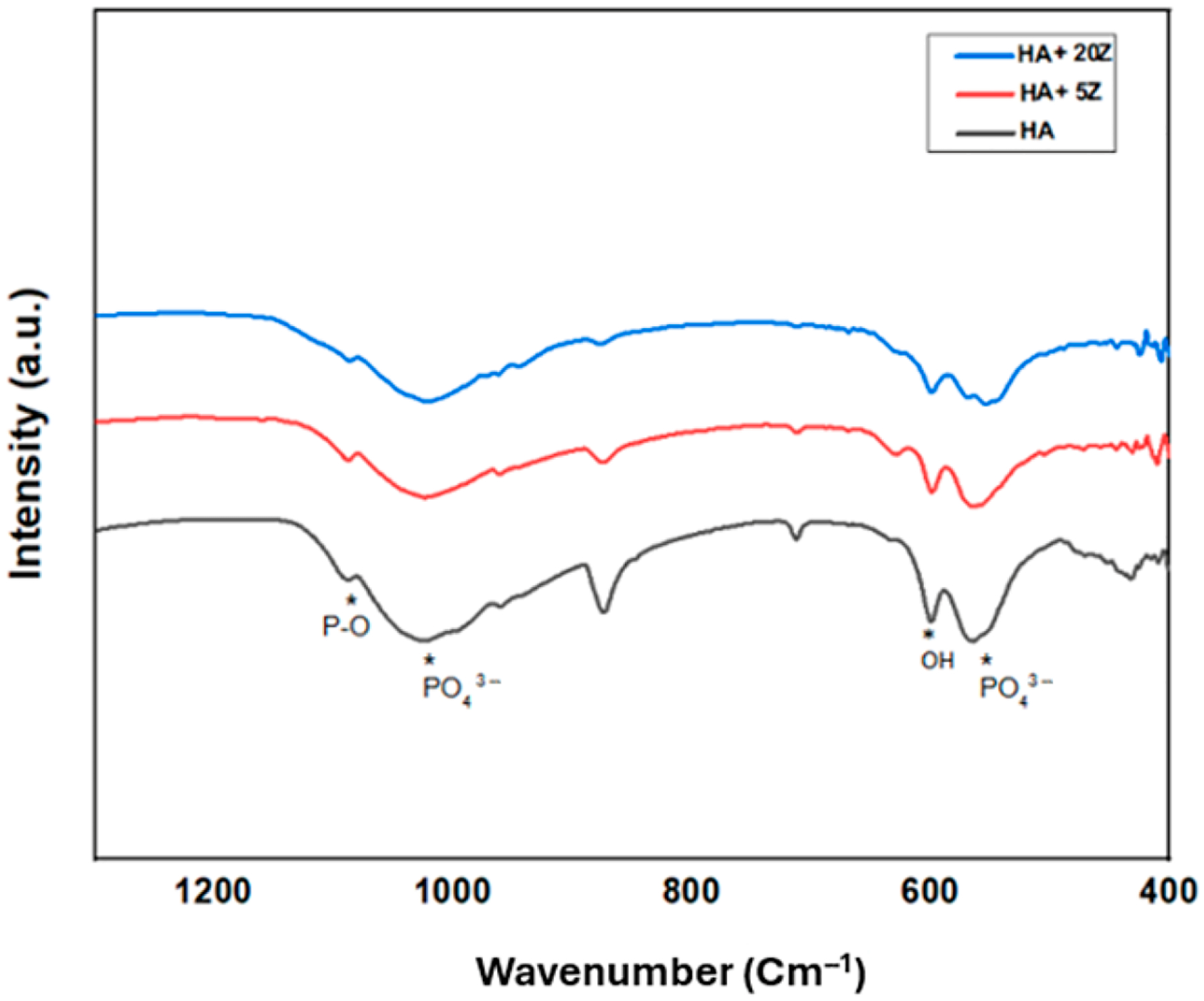
FTIR spectra of the samples show distinct peaks corresponding to PO_4_^3−^ and OH groups within the 1200–400 cm^−1^ range, which are indicated with an asterisk (*) in the spectra. The FTIR results confirm that the HA structure remains intact after adding zirconia.

Figure 3 represents the FTIR spectra of HA-zirconia composite and pure HA within the range of 500 to 1300 cm^−1^. In FTIR, all characteristic peaks for HA are visible. In the region 1090–1020 cm^−1^, the absorption band of P–O is found due to the stretching mode of PO_4_^3−^ groups [40]. The sharp band around the ~550–560 cm^−1^ range represents the bending mode of the PO_4_^3−^ group [40,41]. The vibrational mode of the O–H bond is observed around ~600 cm^−1^ [40].

**Figure 4.**
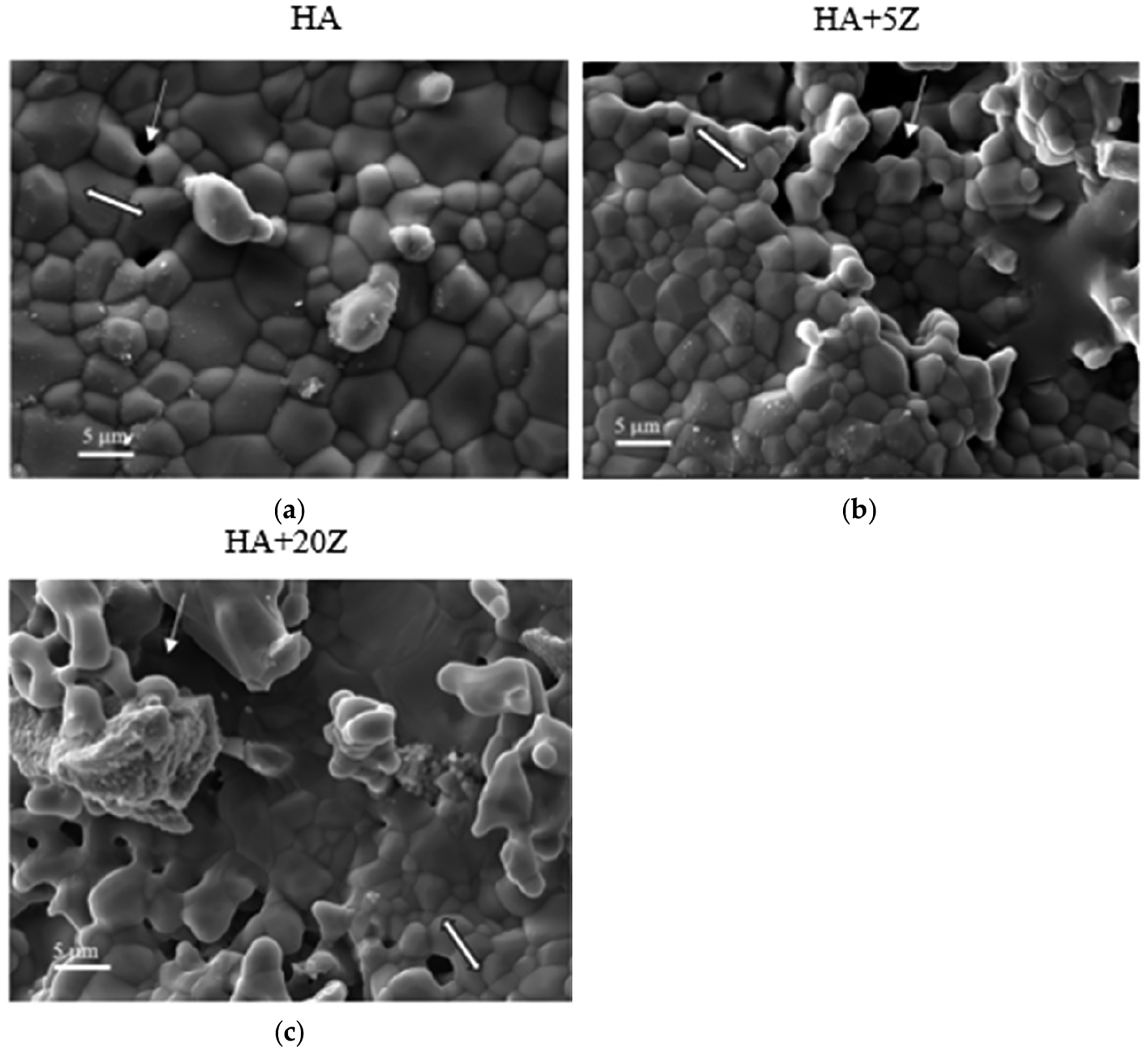
FESEM images showing the surface structure and morphology of the sintered samples: (**a**) HA, (**b**) HA+5Z, and (**c**) HA+20Z. An arrow sign indicates porosity, and a double arrow sign indicates crystalline grains.

**Figure 5.**
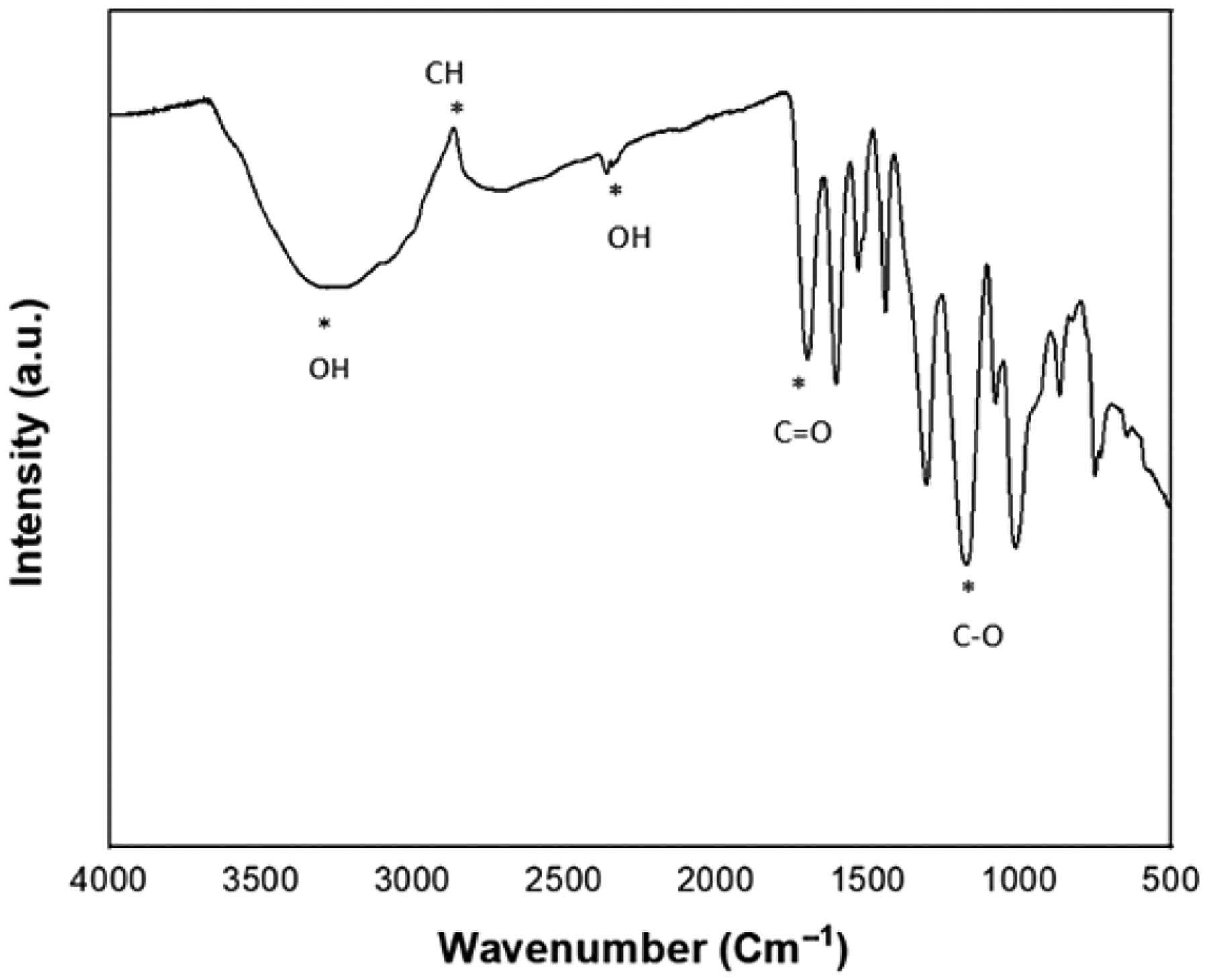
The FTIR spectra of TA within the range of 4000–500 cm^−1^ display characteristic peaks of TA, including C=O, OH, CH, and others, which are indicated with an asterisk (*) in the spectra.

The SEM images of the HA-zirconia composite and only HA samples after sintering are exhibited in Figure 4a–c. Clear grain boundaries are present, which suggests that the entire powder has undergone complete sintering [42,43]. Furthermore, no significantly noticeable change in morphology is observed across the compositions. [14]. Figure 5 demonstrates the FTIR spectra of TA, and the distinct peaks observed correspond to the OH, C-H, C=O, and C-O bonds. At 3275 cm^−1^, adsorption of hydroxyl groups is observed, with an OH stretch detected between 2211–3277 cm^−1^, and adsorption at 2833 cm^−1^ indicates alkane. These spectra are in good agreement with previously published studies [2].

**Figure 6.**
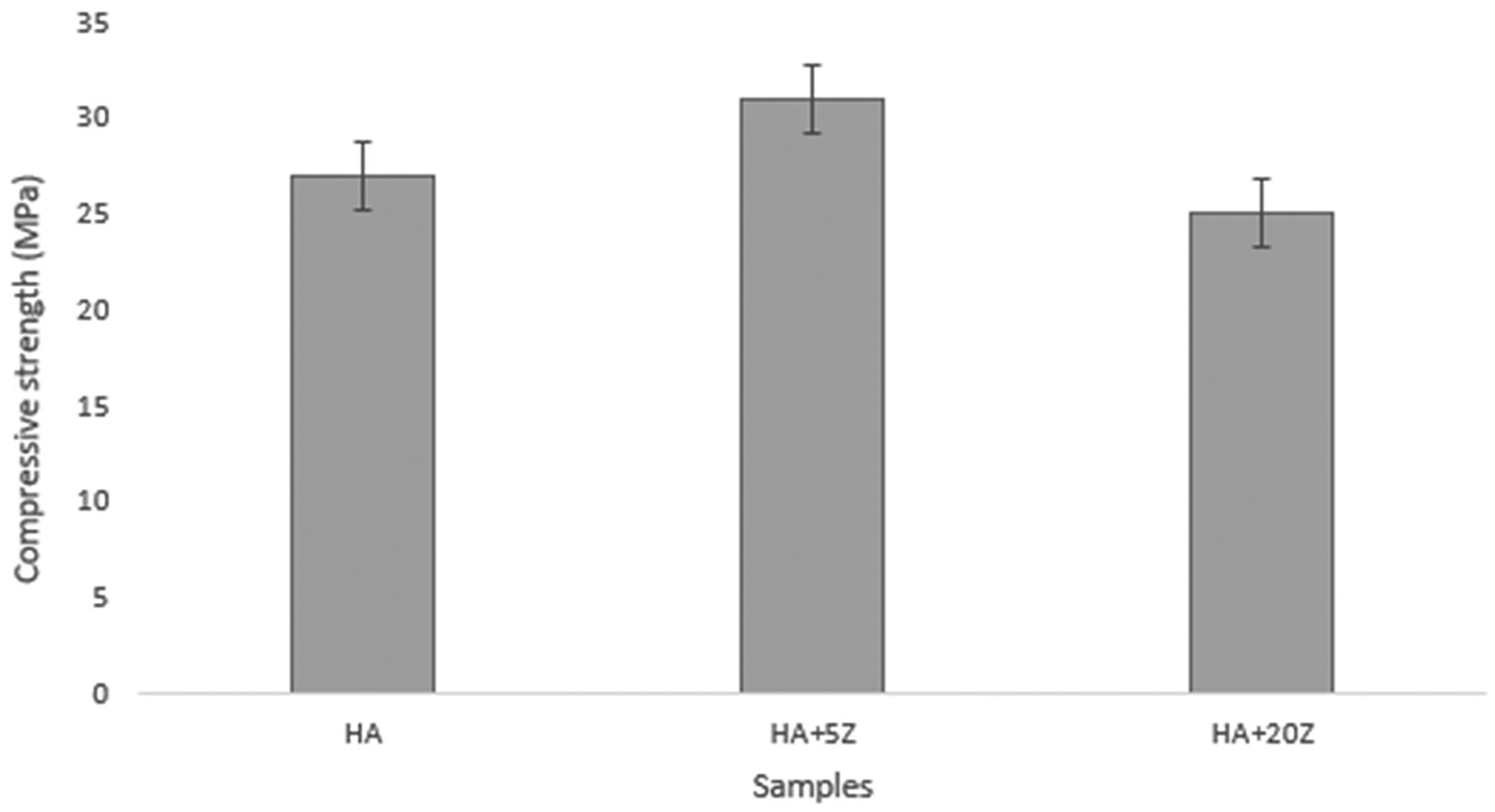
Compressive strength of representative samples showing that 5% HA-zirconia composite have slightly higher compressive strength compared to 20% HA-zirconia composite.

**Figure 7.**
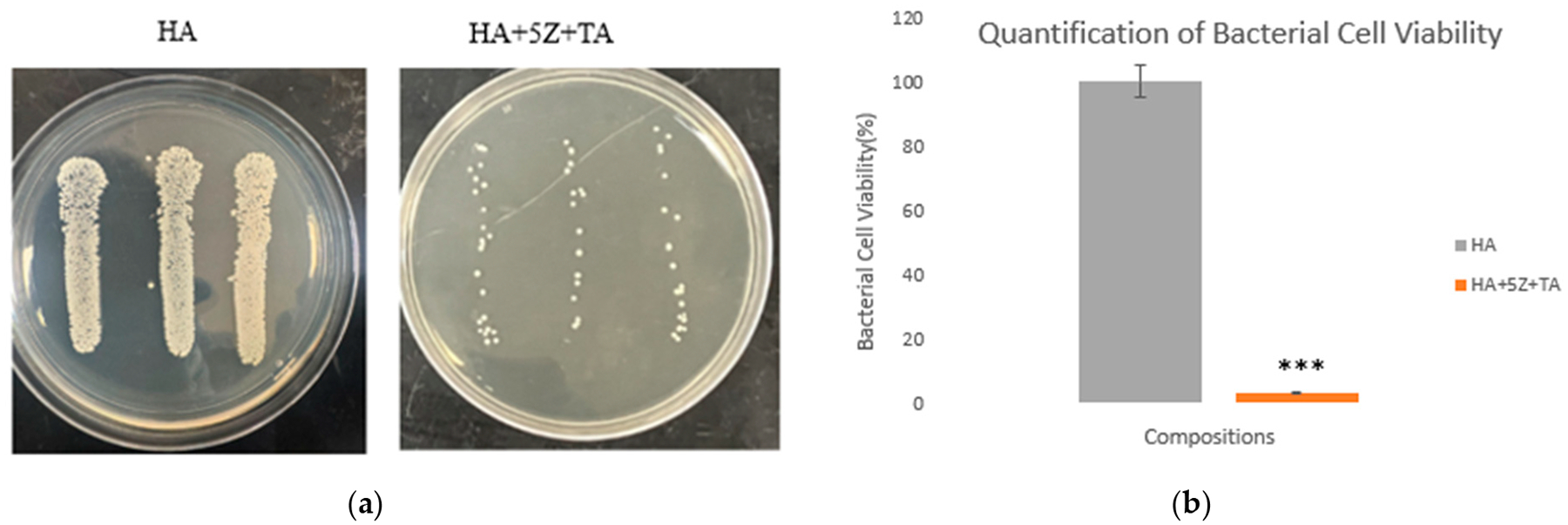
The antibacterial effectiveness was assessed using a modified version of the ISO 22196:2011 standards after 24 h of bacterial exposure to the samples. (**a**) The HA sample shows dense bacterial growth, while the TA-loaded HA-zirconia composite displays a notable decrease in bacterial colony growth on the agar plates. (**b**) Analysis of bacterial cell viability indicates that the TA-loaded 5% zirconia–HA composition achieves up to ~97% antibacterial efficacy, after 24 h of bacterial interaction with the samples (*p* < 0.001 is denoted as ***).

The compressive strength results are shown in Figure 6. The compressive strength value for HA was ~27 ± 2.4 MPa, for 5% HA-zirconia composite, it was ~31 ± 2.1 Mpa, and for 20% HA-zirconia composite, it was ~25 ± 2.9 Mpa. A small decrease in compressive strength was noted for 20% HA-zirconia composite compared to the control sample. The antibacterial efficacy of the tested samples is shown in Figure 7a,b after bacteria loading. The agar plate images (Figure 7a) reveal denser bacterial colonies in the control HA sample, while a notable decrease in bacterial colonies is observed in the treatment tannic acid loaded zirconia sample. The measurement of bacterial colonies (Figure 7b) reveals that the treatment sample with TA-loaded 5% zirconia–HA composite exhibits up to ~97% antibacterial efficacy. TA-loaded HA-zirconia composites lead to a notable decrease in bacterial colonies.

**Figure 8.**
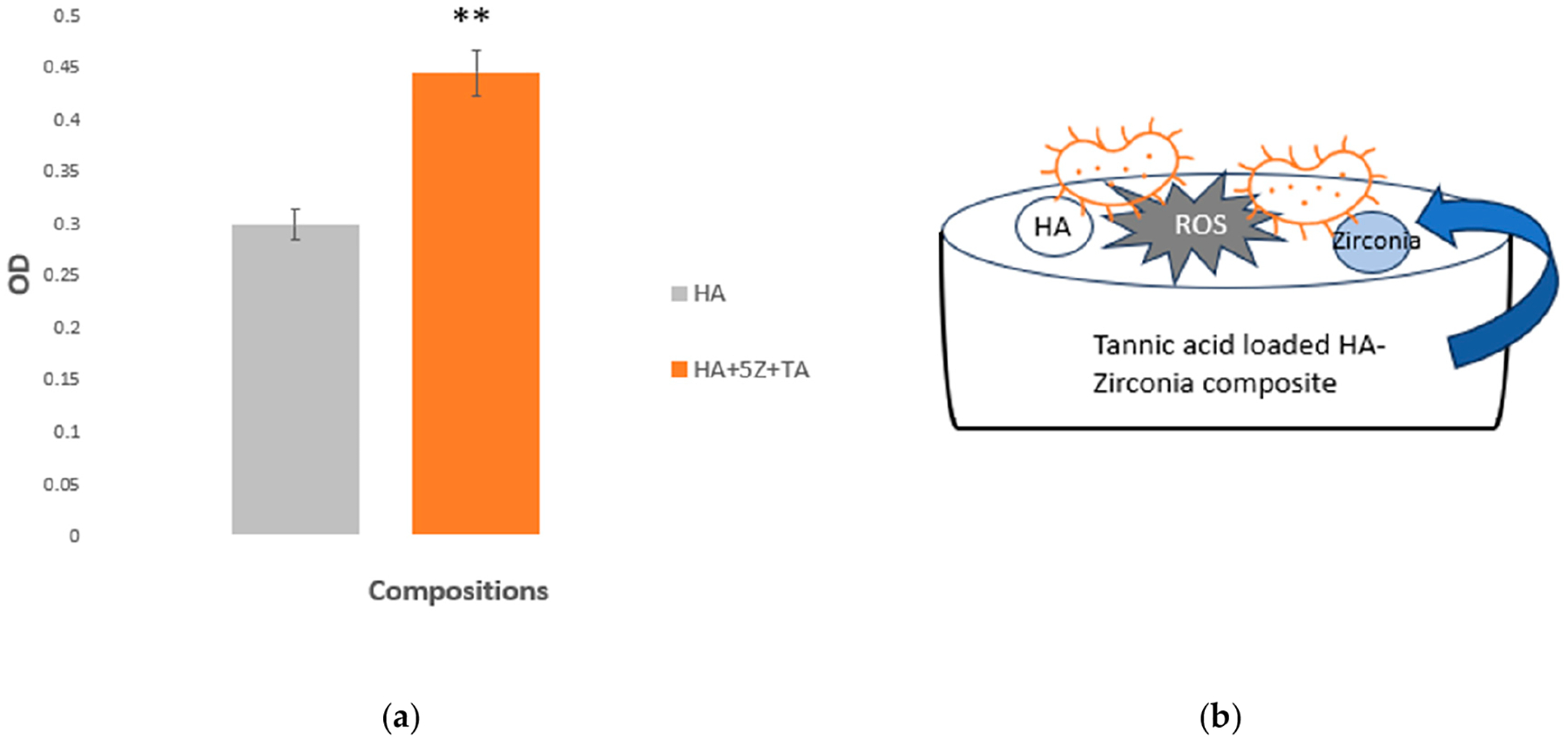
(**a**) The MTT assay results for the tested samples at time point 1 reveal that both HA and HA+5Z+TA exhibit no cytotoxicity. HA+5Z+TA compositions demonstrate higher cell viability compared to the control HA sample. For HA+5Z+TA we got (** *p* < 0.01 is denoted as **). (**b**) A schematic illustrating the antibacterial mechanism attributed to the combined effects of TA and zirconia.

To determine the specific applications of implants, cytocompatibility of biomaterials plays a crucial role. Cytocompatibility testing was performed to demonstrate the biocompatibility of the synthesized scaffolds. The cytocompatibility results of the tested samples at time point one are presented in Figure 8a according to the ISO 10993 standard [41]. At time point 1 (day 2), the TA-loaded 5% HA-zirconia composite showed a higher amount of cell viability compared to the control HA sample. The proposed antibacterial mechanism of TA loaded HA-zirconia composite is shown in Figure 8b.

## 4. Discussion

### 4.1. Natural Medicinal Compounds as an Alternative Drug

The therapeutic efficacy of different natural medicinal compounds in treating clinical disorders is extensively described in ancient Indian medical literature Ayurveda [28]. Current scientific research has elucidated the mechanisms behind the therapeutic potential of many of these compounds. The direct application of these natural medicinal compounds to bone tissue engineering scaffolds is increasingly being explored in scientific studies due to reduced side effects and greater availability of these compounds [28,29]. Our approach involves using TA, an extract derived from tea plants and fruits, which is directly incorporated into HA-zirconia-composite scaffolds as an alternate natural medicine for bone and dental applications. The antibacterial efficacy of TA can further improve the graft’s functionality because TA has a pyrogallol group, which is known to have antibacterial effects against many bacteria, including *S. aureus* [44]. Previous work has shown that TA can create complexes with metals and adhere to surfaces of certain substrates, which makes it desirable in dental implants [45,46]. Furthermore, the built-in antibacterial properties of the scaffolds may help reduce the likelihood of revision surgeries.

#### 4.1.1. HA-Zirconia Composite for Bone Tissue Engineering

Different materials have been tried and tested as potential materials for dental applications [10]. However, these implants are subjected to microbial adhesion, resulting in bacterial infections and biofilm formations that significantly impact the longevity and effectiveness of dental implants and cause implant failure [10]. Moreover, biofilms are more resistant to antibiotics and other treatments, and antibiotics are losing their effectiveness due to antibiotic resistant bacteria. For tissue engineering applications, transition metal ions can significantly improve the biological and mechanical properties and promote bone growth following scaffold implantation. In this work, we have selected zirconia to fabricate HA-zirconia composite because of its bioinert nature. Zirconia is gaining popularity among researchers and is widely used in bone tissue engineering. The phase analysis results, including XRD (Figure 2) and FTIR (Figure 3), for HA and HA-zirconia composite demonstrate the retention of HA phase after zirconia addition. Comparable findings were reported in earlier studies [14,31,35–39].

The radius of divalent Ca^2+^ is 1.00 Å, while the ionic radius of tetravalent Zr^4+^ is about 0.84 Å [5,47]. When HA is mixed with cationic ion zirconia, the zirconium ion replaces the Ca^2+^ ions in the HA structure. Variations in valence ions can lead to changes in crystallite size and crystallinity [48,49]. Previous studies have indicated that substituting smaller cations in HA leads to a reduction in both the crystallite size and the crystallinity of the HA-zirconia composite compared to the only HA [5,41,47]. In this work, the bulk density of 5% HA-zirconia composite is higher than pure HA. The phase analysis results show that the addition of zirconia does not adversely affect the HA phases. This result is consistent with previous studies [35–38,41,47,50].

The microstructural analysis using FESEM (Figure 4a–c) shows a comparable morphology between the HA-zirconia composite and only HA samples. The impact of sintering is evident in the microstructure, where clear grain boundaries are observed without any detrimental effect [14,40,51,52]. Previous studies have examined the impact of zirconia addition on the mechanical properties of HA. [14,15].

#### 4.1.2. Clinical Significance of Antibacterial Scaffolds

Osteomyelitis, or bacterial infection after implantation is a major cause of implant failure and often requires corrective revision surgeries [25,28,53]. Bacterial infections after surgery are caused by *S. aureus* bacteria and alone are responsible for 38% of infections in patients [54]. Currently, antibiotics are commonly used to address this issue, but delivering them directly to the surgical site poses significant challenges, and the rise of drug-resistant bacteria adds further complexity to the treatment process [55]. Our study examined the antibacterial efficacy of TA-loaded HA-zirconia composite against *S. aureus*, the primary bacteria responsible for osteomyelitis. The results (Figure 7a,b) demonstrate the significant antibacterial efficacy of HA-zirconia composite when combined with tannic acid (TA), achieving up to ~97% antibacterial efficacy in the case of 5% HA-zirconia composite, against *S. aureus*, compared to the control. The evaluation of antibacterial efficacy after directly incorporating TA into HA-zirconia composite represents a novel aspect of this research. TA exerts its antibacterial effect by penetrating the bacterial cell wall, disrupting metabolism, and inducing cell death. It simultaneously inhibits the uptake of sugars and amino acids to restrict further bacterial growth [23,56]. The antibacterial test results (Figure 7) show that the presence of TA, along with zirconia, significantly reduces bacterial colony formation when compared to the control group. The MTT assessment is illustrated in Figure 8a. The synergistic effects of the zirconia and TA substantially improve NIH3T3 cellular viability compared to the control, making TA-loaded HA-zirconia composite a potential material for diverse tissue engineering applications.

#### 4.1.3. The Function of Tannic Acid Loaded HA-Zirconia Graft

TA possesses a variety of therapeutic benefits, such as antioxidant, antimutagenic, and antitumor effects, and is also recognized for its homeostatic properties and chemo-preventive potential [57]. Due to its bioactive qualities, TA is being explored as an organic polymer additive to improve the characteristics of materials for biomedical applications and is commonly used in nutritional products and other consumables [58–60]. The FTIR analysis of TA’s functional groups (Figure 5) aligns well with previous studies [2,61]. Transition metal oxide zirconia, demonstrates antibacterial properties due to its strong oxidative nature, which generates reactive oxygen species (ROS) that kill bacteria and prevent adhesion on surfaces containing Zr^4+^ [62]. The Zr^4+^ ions disrupt bacterial cell membrane permeability, affect amino acid metabolism, and interact with proteins, nucleic acids, and enzymes, inhibiting bacterial growth [62–64]. These ions also interfere with specific bacterial metabolic pathways, ultimately causing cell death [62–64]. The combination of TA’s antibacterial properties and zirconia’s ability to produce reactive oxygen species (ROS) creates a powerful antimicrobial effect. The integration of TA and zirconia into HA could significantly reduce the formation of bacterial biofilms on dental materials, leading to better long-term outcomes.

#### 4.1.4. Contributions to Science and Direction to Future Research

The key scientific contributions of our study are as follows: (1) the development of a natural medicinal compound, TA-loaded HA-zirconia composite, as a novel scaffold for bone tissue engineering, and (2) the improvement of the antibacterial properties of HA-zirconia composite due to the presence of TA, particularly against *S. aureus*, the major bacteria that causes osteomyelitis. Future research can focus on more biological studies with this alternative system, as well as exploring its potential for *in vivo* bone regeneration applications.

## 5. Conclusions

Our study demonstrates successful HA-zirconia composite fabrication, along with enhanced antibacterial properties. The assessment of comprehensive physical and morphological characterization, including XRD, FTIR, FESEM shows no adverse effect in HA as a result of zirconia addition. Zirconia addition with HA followed by tannic acid loading enhances the antibacterial properties of the composite. Tannic acid-loaded HA-zirconia composite showed ~97% antibacterial efficacy against *S. aureus* and acceptable mechanical properties. The optimal amount of zirconia addition, does not adversely affect the HA matrix. NIH3T3 cell culture results indicate that tannic acid loaded HA-zirconia composite is biocompatible and could be used in tissue engineering for bone repair. Our findings highlight the effective applications of zirconia, tannic acid, and HA for bone and dental applications. Thus, we can conclude that HA-zirconia composite loaded with tannic acid appears to be a potential candidate for bone tissue engineering, offering enhanced antibacterial properties without compromising the cytocompatibility. However, further research is needed to fully explore the biomedical applications of these unique bioceramic materials.

## Figures and Tables

**Table 1.** The bulk density (g/cm^3^), volume, radial, and longitudinal shrinkage (%) after sintering, respectively.

Sample ID	Bulk Density (g/cm^3^)	Volume Shrinkage (%)	Radial Shrinkage (%)	Longitudinal Shrinkage (%)
HA	2.66	55.67	23.75	21.78
HA+5Z	2.72	57.77	24.97	24.48
HA+20Z	2.03	42.73	16.96	16.39

## Data Availability

The original contributions presented in this study are included in the article. Further inquiries can be directed to the corresponding author(s).
